# Optimizing Polymer Infusion Process for Thin Ply Textile Composites with Novel Matrix System

**DOI:** 10.3390/ma10030293

**Published:** 2017-03-15

**Authors:** Somen K. Bhudolia, Pavel Perrotey, Sunil C. Joshi

**Affiliations:** 1 School of Mechanical and Aerospace Engineering, Nanyang Technological University, 50, Nanyang Avenue, Singapore 639798, Singapore; mscjoshi@ntu.edu.sg; 2 Institute for Sports Research, Nanyang Technological University, 50, Nanyang Avenue, Singapore 639798, Singapore; PPERROTEY@ntu.edu.sg

**Keywords:** NCFs, VARI, manufacturing, Elium^®^, thermoset, thermoplastic, optimisation

## Abstract

For mass production of structural composites, use of different textile patterns, custom preforming, room temperature cure high performance polymers and simplistic manufacturing approaches are desired. Woven fabrics are widely used for infusion processes owing to their high permeability but their localised mechanical performance is affected due to inherent associated crimps. The current investigation deals with manufacturing low-weight textile carbon non-crimp fabrics (NCFs) composites with a room temperature cure epoxy and a novel liquid Methyl methacrylate (MMA) thermoplastic matrix, Elium^®^. Vacuum assisted resin infusion (VARI) process is chosen as a cost effective manufacturing technique. Process parameters optimisation is required for thin NCFs due to intrinsic resistance it offers to the polymer flow. Cycles of repetitive manufacturing studies were carried out to optimise the NCF-thermoset (TS) and NCF with novel reactive thermoplastic (TP) resin. It was noticed that the controlled and optimised usage of flow mesh, vacuum level and flow speed during the resin infusion plays a significant part in deciding the final quality of the fabricated composites. The material selections, the challenges met during the manufacturing and the methods to overcome these are deliberated in this paper. An optimal three stage vacuum technique developed to manufacture the TP and TS composites with high fibre volume and lower void content is established and presented.

## 1. Introduction

Carbon Fibre Reinforced Plastics (CFRPs) composites offer higher specific properties compared to other classes of composite materials. Manufacturing and properties of the overall composite system is a function of two building blocks, i.e., fibres and the matrix. Thus, choosing a right mix of fibres and matrix system is of paramount importance. Regarding fibres, usually the woven carbon fabrics are very appealing and continuously used owing to ease of handling, processability using liquid infusion methods and higher specific properties. However, there is localised loss of mechanical properties due to associated crimps which are inherent to these types of fabrics [1]. Non Crimp Fabrics (NCFs) made of several dry UD plies stitched together offer new possibilities in processing as several plies can be stacked at once. In addition, it reduces resin rich areas and stress concentrations that can be found in woven fabrics [2]. NCFs can be combined with the use of spread tows to manufacture so called thin plies that have fibre areal weight (FAW) lower than 150 g/m^2^ and that can go down to 30 g/m^2^ (up to 1/6th of the standard ply thickness) [3,4]. These thin NCFs have potential to significantly reduce the delamination in the laminated composite structures [3]. Thermoset resins are largely used because of their enhanced mechanical properties and medium curing temperature range. Thermoplastic resins are rarely used as they usually require high temperature processing [5,6]. However, thermoplastics have properties that could benefit composite parts greatly such as their high fracture toughness [7], good vibration dampening capacities [8], post formability [9], high impact resistance and, importantly, the recyclability [10,11]. Even though there is literature available on the properties of composites with reactive processing thermoplastics such as Polyamide 6 (PA6), Polyurethanes (PU), Cyclic Butylene Terephtalate (CBT) and many more, they require very high temperature processing [12,13,14]. Ma and Chen [15] studied the properties of PMMA based composites manufactured using pultrusion process with viscosity range of 1000–1200 cP and require adequate temperature during the casting phase.

Majority of composites products in various applications ranging from automotive, aerospace, sporting, marine and many more are manufactured using prepreg processes. The process of prepreg moulding using autoclave or hot presses results in relatively high mechanical properties and high fibre volume fraction and thus meets the requirements of light and stiff products [16]. The process, however, is labour intensive and costly [17]. Efforts are therefore required to offer an alternative to the prepreg process. Over the years, VARI process has become a versatile method for processing thermoset and thermoplastic resin having low viscosity, if used by controlling the process parameters such as vacuum and proper degassing [18,19,20]. Studies have also been carried out on infusion processes for composites with polypropylene [21] and PETI-RFI, a polymer showing thermoplastic characteristics and cross links at higher temperature [22]. This process is serving many industries from sports to light rail to marine to aerospace [20]. Competition sail boats, hulls and decks, armours, car seats and even cargo doors in aircrafts are now manufactured using vacuum assisted resin infusion (VARI) process, which enables cost effectively producing large parts with relatively good mechanical properties. The manufacturing of NCFs via resin infusion process is not an easy subject to deal with because of the different stitching patterns and resistance it offers to resin flow, especially the thin variants of NCFs [23,24]. The VARI parameters, which require optimisation, include vacuum level, resin flow speed, temperature and infusion time, while material parameters include fibres, resin, flow mesh and peel ply. Note that for each fibre/resin system and product geometry, the process and material parameters need to be optimised. There is no detailed study recorded regarding liquid processing for NCF thin plies for thermoplastic resin using VARI or allied processes curable at room temperature. As carbon fibre thin plies and Elium^®^ 280 resin possesses tremendous potential to be a suitable material for structural composite applications owing to their combined mechanical and dampening performance, their manufacturing optimisation holds key in terms of quality, ease, and time and energy saving. The scope of this paper is to present a way of processing dry carbon NCF thin C-Ply™ using the VARI process to understand the flow behaviour while impregnating this grade of fibres. Two different resin systems are used for comparison, thermoset resin for benchmarking and the thermoplastic resin, both suitable for liquid processing. The chosen epoxy resin is EPOLAM 5015/5015 from Axson having a comparable mixed viscosity of 210 cP as that of Elium^®^ 280, a first of its kind liquid MMA based thermoplastic resin possessing 200 cP viscosity and can be processed using the VARI and allied process at room temperature. The effect of process parameters such as vacuum level, infusion time and the optimal usage of flow mesh during the manufacturing of both thick C-Ply™ /TP and thin C-Ply™/TS have been studied in detail. A manufacturing strategy with optimised process parameters after manufacturing and charactering 82 panels is proposed which promises to achieve a high fibre volume fraction and low void content composite panels. Manufacturing strategy is developed for Elium^®^ 280 resin to be used with thick NCFs and thin NCFs, while the same technique is generalised for epoxy composites with many trials undergone using the same fabrics but with a thermosetting epoxy resin that is comprehensively used in liquid infusion processes.

## 2. Materials and Methods

### 2.1. Materials

The reinforcement material used in current study is thin carbon NCF bi-angle ply C-Ply™ (≤150 g/m^2^) and thick carbon NCF bi-angle C-Ply™ (≥150 g/m^2^) from Chomarat In addition, woven glass fibres acquired from Polymer Technologies, Singapore have been used in the current investigation. Table 1 gives the details (FAW, and orientations) of glass fibres, epoxy (TS) and thermoplastic (TP) sized bi-angle C-Ply™. It should be noted that the C-Ply™ with different orientations are specially sized to be compatible with TP and TS matrices.

**Figure 1:**
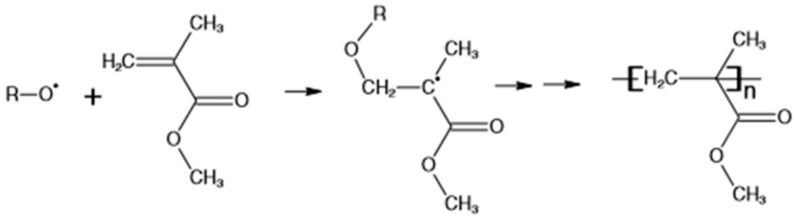
Free radical polymerisation of Methyl methacrylate (MMA) monomer to Poly Methyl methacrylate (PMMA) polymer with peroxide initiator [13].

Recently invented liquid thermoplastic resin, Elium^®^ 280 (reactive liquid MMA) from ARKEMA, is used as the thermoplastic solution. Elium^®^ 280 resin can be cured at room temperature with demoulding time of 3.5 h, which includes 2 h of post-curing at 80 °C, and promises to be suitable solution for manufacturing composite parts while matching the mechanical performance of its counterpart thermoset resins. At higher temperature of 80 °C, Elium^®^ 280 resin cures in 6 min. Demoulding time is also considerably reduced as the post curing step is not required. Elium^®^ resin being in liquid state possesses viscosity of 200 cP and is very comparable to the mixed viscosity of Epolam 5015/5015 epoxy resin which is used as a baseline comparison in current investigation. It undergoes radical polymerisation from its monomer MMA to its polymer PMMA with peroxide initiators (see Figure 1) [13]. Table 2 can be referred for the data sheet properties of the chosen resin systems in current work acquired from the suppliers of the resin. It should be noted that the Elium^®^ 280 has advantage with six times faster demoulding time and can operate at a much higher temperature range with Tg of 120 °C, even though it is a room temperature cure resin.

### 2.2. Vacuum Assisted Resin Infusion (VARI) Process

**Figure 2:**
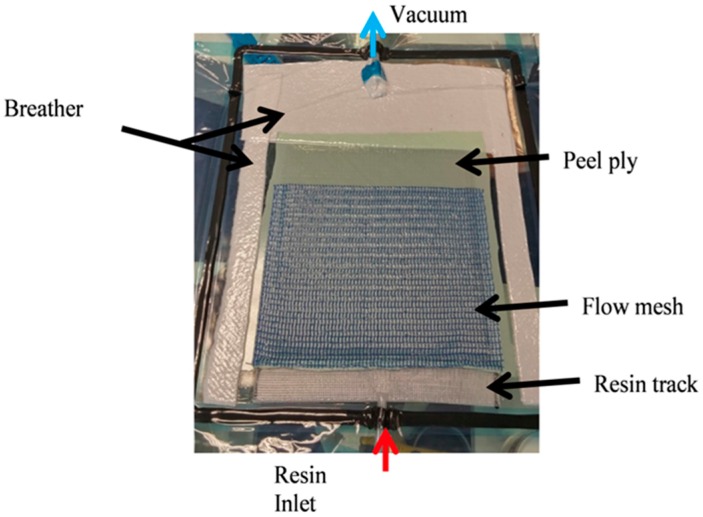
Vacuum assisted resin infusion (VARI) set-up.

A resin infusion kit was used which included peel plies, breather, resin inlet and vacuum outlet hoses, pressure pot, flow mesh, etc. to facilitate the VARI process. The fabrication process for manufacturing laminates started with cutting dry C-Ply™ (kept in a dry cabinet at 50% relative humidity) into the desired dimensions (length × width). The bi-angle C-Ply™ was stacked upon each other to make a laminate of particular lay-up. Laminates were weighed after cutting to keep account of dry fibres to be infused which will be an important parameter later to calculate the fibre volume fraction (V_f_). Mould plate was first cleaned and then waxed with Chemlease^®^ 41-90EZ release agent, so that proper demoulding can be achieved after curing along with a glossy surface finish. It should be noted that at least four layers of wax were first applied on the plate before first infusion, later for subsequent infusions, applying two layers was sufficient. Weighed fibres were laid on the waxed mould followed by peel ply and flow mesh. While the function of coated nylon peel ply (Bleeder lease B from Airtech) was to achieve a textured surface that can absorb excess resin, flow mesh (Green flow 75 from Airtech) was used to promote the resin flow. Breather on the side and at the end of the set-up was used to ensure that air is properly sealed under vacuum and also to absorb the excess resin. After fibres, peel ply, flow mesh and breather were arranged, the whole set up was vacuum bagged, as shown in Figure 2. Resin track was also used to ensure the linear flow front.

After the set-up was done, the resin inlet was locked using clamps and the hose from the vacuum pump was attached to the outlet of the set up via the pressure pot. It should be noted that first a near full vacuum was used before the infusion to remove all the air trapped in the laminate (31.3–90 mbar). Then, after keeping the pump off, leak test was carried for at least 10 min to check the feasibility of the fabrication set up and ensure that there were no leaks which can later create voids in the cured laminate. Once the leak test was passed, it was practiced to debulk the fibres by releasing the vacuum and again putting it back and the process was repeated for at least four times. This leads to the proper compaction of fibres and ensure consistent fibre volume fraction and thickness. Once the whole set up was ready to be infused, the next step was the resin preparation. For Epolam 5015/5015 resin, hardener fraction was 100:30, while, for Elium^®^ 280 thermoplastic resin, 2.3% to 3% of peroxide was used as used as polymerizing agent. Resin and hardener were mixed properly using stirrer in PE or PP cup. Later, degassing of epoxy resin was carried out at 90 mbar vacuum for 20 min using the resin degassing chamber. The Elium^®^ 280 resin infusion time was only 40 min at room temperature, so degassing was not recommended and air bubbles were controlled by playing with inlet flow speed and adding a cap on the top of the resin recipient during infusion. Then, the resin was infused and the flow front was marked and recorded.

### 2.3. Fibre Volume Fraction Calculation

Fibre volume fraction is always an important parameter which reflects on the quality of the composite laminates manufactured. The fibre volume fraction was calculated using Equation (1) [25],

(1) $$V_{f}=\;\frac{1}{\left(1+\frac{\rho_{f}}{\rho_{r}}\left(\frac{1}{w_{f}}-1\right)\right)}$$

where, $w_{f}=\mathrm{fibre}\;\mathrm{weight}\;\mathrm{fraction},\;\rho_{f}=\mathrm{density}\;\mathrm{of}\;\mathrm{fibre}\;\mathrm{and}\;\rho_{r}=\mathrm{density}\;\mathrm{of}\;\mathrm{resin}$.

The fibre volume fractions achieved consistently for thin C-Ply™/Elium^®^ 280 are 52%–60% and C-Ply™/Epolam 5015/5015 are 58%–62%. Since the epoxy resin has a longer gel time compared to the Elium^®^ 280 resin, more time for getting the excess resin out of the laminate helped to increase V_f_.

### 2.4. Void Estimation

Quantitative estimate of void content was carried out using ASTM D792 [26] and ASTM D2734-09 [27]. Many researchers have used these test standard method to quantify the void content [28,29]. ASTM D792 method was used to calculate the composite measured density while ASTM D2734-09 was used to calculate composite theoretical density and void content. Measured density (**M**) of the composite laminate was calculated using Equation (2) [26].

(2) $$M=\left(\frac{a}{\left(a-b\right)}\right)\times density\;of\;water\;or\;propanal$$

where **a** is weight of laminate in air (g), **b** is weight of laminate in water/propanol (g) and density of propanol = 0.785 g/cm^3^.

The theoretical composite laminate density and void content were calculated using Equations (3) and (4), respectively [27].

(3) T=100/(Rρr+rd)

where, **T** = Theoretical composite density, **R** = Weight % of resin in composite, $\rho_{r}$ = Density of resin, **r** = weight % of reinforcement and **d** = density of reinforcement.

(4) $$V=\frac{100\left(T-M\right)}{T}$$

where, **V** is void content (%), and **T** and **M** are theoretical and measured composite densities, respectively (g/cm^3^).

The void contents for C-Ply™/Elium^®^ 280 laminates were found to be consistently in the range of 0.1%–1%, while for C-Ply™/Epolam 5015/5015, the void content was between 0.5% and 1.8%. Considering, no substantial pressure was applied as in case of conventional autoclave or hot press curing, the void content of panels manufactured using VARI technique were found to be satisfactory.

### 2.5. Interlaminar Shear Strength Test (ILSS Test)

ASTM D2344 [30] standard was used to perform this test. The short beam test was used to determine the interlaminar shear strength of fibre reinforced composite. Basically, the test is similar to a three-point bending test with a very small specimen to maximise shear in front of tension or compression while bending. A span/thickness ratio of 5 was used which resulted in the gauge length of 10 mm. Interlaminar shear strength was calculated using Equation (5) [30],

(5) SH=0.75×PBS

where **S_H_** is the shear strength (N/mm^2^), **P_B_** is the breaking load (N) and **S** is the sectional area of the specimen (mm^2^).

## 3. Results and Discussion

### 3.1. Key Considerations for Infusing NCFs with TP and TS Matrices

This section deals with some of the important findings which should be considered before injecting NCFs with TP and TS matrices. The challenges and methods to overcome them are also discussed in this section.

#### 3.1.1. Deactivation of Initiator

**Figure 3:**
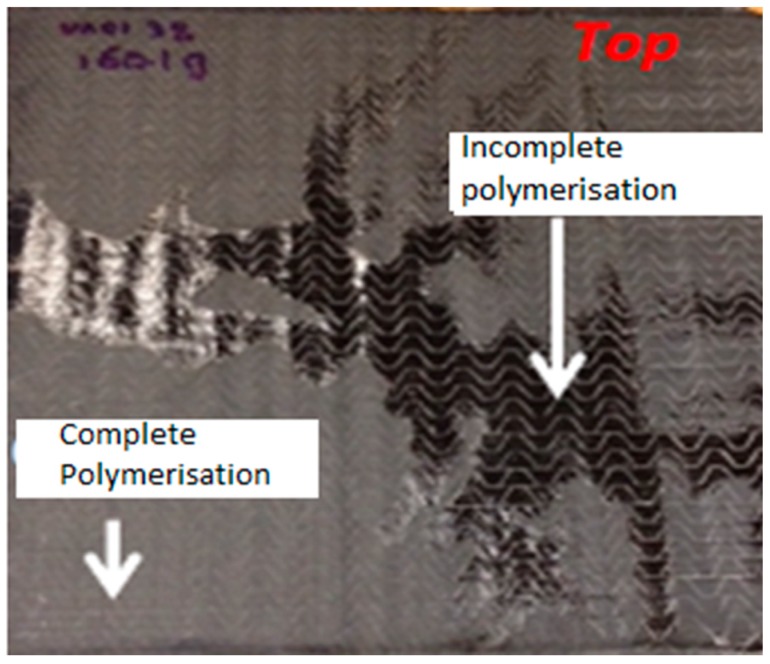
Incomplete polymerisation due to deactivation of initiator.

It was noticed during manufacturing trials that either due to the presence of the moisture in the fabric or in some cases the water based peroxide may lead to inconsistent resin polymerisation if corrective measures were not taken. A two-step process was adopted to ensure that the fabric is moisture free before the use. First, it was stored in the dry cabinet due to very humid environment in work place and just before the infusion, fabric was dried in a hot press under vacuum for 15 min. Figure 3 shows the laminate with incomplete polymerisation in some parts of the panel. Another important reason for deactivation of initiator is the presence of dust particles on the fabrics which locally disturbs the anionic polymerisation during the resin infusion process [13,31]. In current research, it was ensured that the fabrics were free from the possible dust particles by carrying out the processing in a clean room environment.

#### 3.1.2. Effect of Low Permeability of Thin NCFs

Low permeability of these fabrics restricts the polymer flow inside the mould during the infusion process. Usually the polymer flow through the fabric media is governed by Darcy’s Law, which in 1-D is written as Equation (6) [32,33],

(6) $$\frac{Q}{A}=\frac{k}{\eta }\frac{\Delta p}{L}$$

where ***Q*** is resin consumed (m^3^/s), ***A*** is the cross sectional area of flow (m^2^), ***k*** is the permeability of fabric preform (m^2^), η is the viscosity of the resin (Pa-s), Δp is the pressure difference between inlet and outlet of the resin tubes (Pa) and ***L*** is the progressed flow front (m).

It is clearly observed from Equation (6), lower permeability of fabric preform (lower k value) hinder the polymer flow significantly. Thick NCFs have higher in- and out-of- plane permeability than the thin NCFs and hence the infusion process is completed much earlier. Thin NCFs used in current investigation are manufactured using spread tow thin-ply technology where dry ply weight of less than 100 g/m^2^ is achieved. In the process, flat fabrics of low thicknesses are achieved by mechanically spreading the larger tows, such as 12 k, 24 k, etc. [3]. Low thickness tapes are stitched to an adjacent orthogonal fabric with no associated crimp and hence have different fabric arrangements than the regular tow, as in the case of thick NCFs. As in-plane permeability is the function of fibre radius, spread tow fabrics have significantly lower permeability compared to thick plies [24]. Considering the preform of similar thicknesses, thin NCF preform has double the number of plies which further increases the infusion time. Table 3 shows the permeability values for thin C-Ply^TM^ received from the supplier of the NCFs, Chomarat. While the values for thick NCF (400 g/m^2^) was not available, in-plane permeability values for the similar higher areal weight fabric found from the literature are reported for comparison. Out-of plane permeability for Seartex thick ply (at same V_f_) was not available. It was noticed from the available data that higher areal weight fabrics have comparatively lower through the thickness permeability. As can be seen from Table 3, for thin NCFs, K_z_ is much lower than K_x_ and K_y_, while the in-plane permeability of thicker plies are higher compared to thin variants. Usually, there is the detrimental effect of mould temperature on the process cycle, but both the TP and TS resins used in current research cure at room temperature. In addition, it is important to mention that the resin infusion should be carried out as soon as possible after mixing to avoid homopolymerisation, which significantly affects the infusion window.

#### 3.1.3. Constraint on Resin Degassing

**Figure 4:**
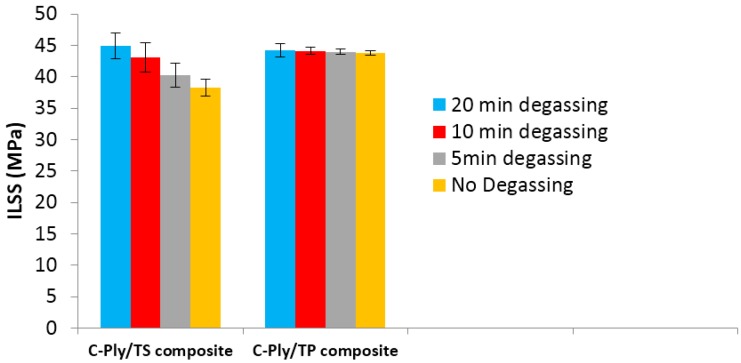
Effect of resin degassing on Interlaminar Shear Strength (ILSS) test properties of C-Ply^TM^ composites.

While there was no considerable issue in resin degassing for epoxy resin due to very long gel time, the resin degassing was carried out for 20 min before infusion. Degassing was found to significantly improve the interlaminar shear strength (ILSS) of the epoxy composite laminate. However, for the liquid MMA, Elium^®^ 280 resin degassing is very tricky due to very low infusion time of 40 min at room temperature for current variant of Elium^®^ 280 resin used in present investigation. Interestingly, for Elium^®^ 280 resin to be cured at room temperature, in situ or in mould degassing was done by clamping more or less the resin pipe a few times during the first few minutes of infusion and was found to be sufficient to manufacture a low porosity laminate (<1%). The effect of degassing on the quasi-isotropic C-Ply^TM^/TS and C-Ply^TM^/TP laminates are shown in Figure 4. Five specimens were tested for each laminate configuration with different degassing times. It was noticed that amount of resin degassing time does not significantly affects the ILSS properties for Elium^®^ 280 composites and even 5 min degassing time was found sufficient to get the optimal ILSS properties along with reduced porosities. The void content for Elium^®^ 280 composite laminates for all the instances of degassing were found to be <1%. The degree of conversion was also not affected significantly, which generally reduces the strength and modulus of the composite laminates. In the case of epoxy composites, the amount of resin degassing was directly proportional to the void content. The epoxy composite laminates with 5, 10, 15 and 20 min degassing have 12.3%, 4.3%, 2.7% and 1.01% void content, respectively. Porosities due to lower degassing time is the major cause for the premature interlaminar shear failure of epoxy composites [35].

#### 3.1.4 Effect of Initiator on Degree of Conversion

Benzoyl peroxide (BPO) is used as an initiator for polymerisation of Elium^®^ 280 resin. Standard 3% was used as the optimal percentage because it leads to desired degree of conversion, which is required for a polymer to have high strength, stiffness and chemical resistance. It should be noted that the BPO percentage can be varied between 2% and 3%. However, as the lower percentage will certainly increase the peak time for polymerisation, there will be some degree of compromise in the total degree of conversion. Usually, high degree of conversion is achieved with 3% BPO at room temperature.

### 3.2. Infusion Process Optimisation

**Figure 5:**
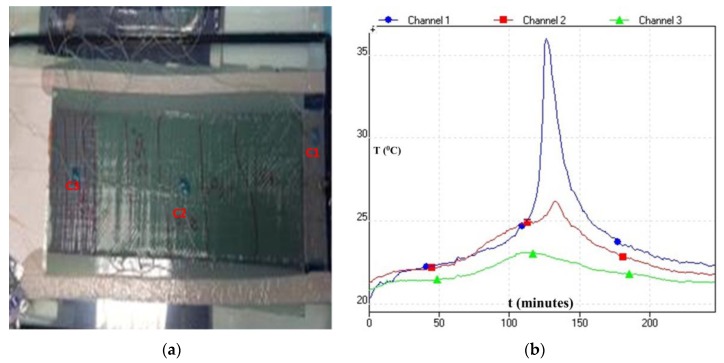
Thermoplastic resin infusion set-up showing (**a**) mounted thermocouples (**b**) t-T curve recorded during the infusion.

The current development deals with a cost-effective approach of manufacturing composites laminates using VARI technique, whereby a room temperature cure epoxy and a thermoplastic resin system are used to infuse thick and thin carbon NCFs. The manufacturing with NCF has not been easy: because of its stitching pattern, wettability and intrinsic resistance it offers to the polymer flow, the flow and curing characteristics of the polymer also play a vital role [23,24,36,37]. Furthermore, especially for Elium^®^ 280 resin, the process optimisation is critical as free radical polymerisation of methyl methacrylate (MMA) monomer from its polymer PMMA form is tricky as there are chances that the exotherm of the monomer may reach and resin start to boil due to uncontrolled vacuum which generally results in much higher void content [13]. Thus, an incremental study was performed to investigate the infusion of NCF thin and thick C-Ply™ with TP and TS resin systems. More than 80 panels (up to 300 mm × 480 mm) are manufactured in this research to optimise the process parameters. For Elium^®^ 280 resin infusion, thermocouples were mounted on the surface of the panel to record the temperature exotherm as shown in Figure 5. USB TC-08 Thermocouple Data Logger and type K thermocouples were used for this purpose. When the temperature reaches its peak and falls down sharply, it signals the complete polymerisation and demoulding can be carried out immediately. Various process parameters were needed to be optimised for complete impregnation of NCF panels with consistent fibre volume fraction. The following subsections deal with the details of the various optimised parameters.

#### 3.2.1. Mould Characteristics

**Figure 6:**
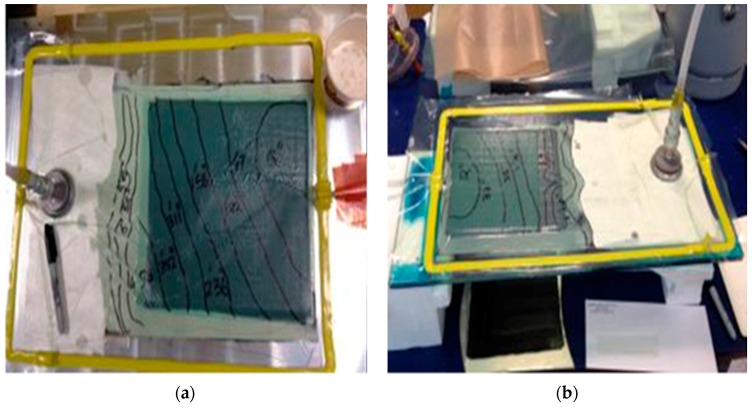
Vacuum Assisted Resin infusion set-up using: (**a**) Al plate; and (**b**) glass plate with a mirror below to trace the flow evolution.

Aluminium (Al) plate was used as a mould material and no anomalies were observed in infusing woven glass and carbon fabrics. The usage seemed fine for standard woven fabric but not for thin C-Ply™ NCFs, where the resin flow front in the plane was faster than through the thickness direction. While infusing NCFs with both the matrices, the bottom of the panel (e.g., thin C-Ply™, (+45/−45)_4S_, 1.7 mm), was not fully infused from the bottom when it was demoulded. A glass plate combined with the use of a mirror enabled tracing the evolution of the resin flow front from both the top and bottom faces of the laminate as depicted in Figure 6b. The glass plate being smoother improves the panel surface finishing and facilitates easier demoulding. It also dissipates the heat faster during in situ polymerisation in the case of thermoplastic resin infusion.

#### 3.2.2. Flow Mesh

**Figure 7:**
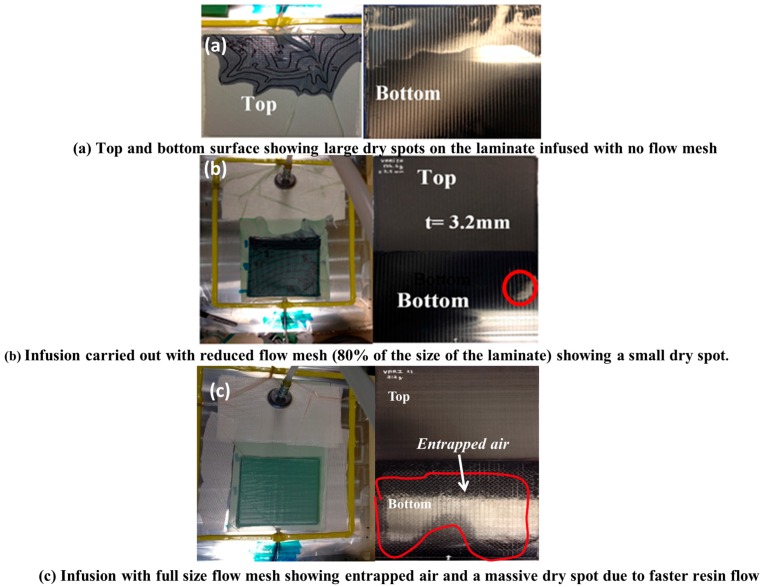
Evolution of VARI process optimisation with different flow mesh length. (**a**) No flow mesh; (**b**) 80% flow mesh; (**c**) full size flow mesh.

The carbon NCF was first infused with full flow mesh (same as the size of the panel), as seen in Figure 7a. However, the full flow mesh accelerated the flow in the longitudinal direction, the panel was not fully infused in the thickness direction, and a massive dry spot with entrapped air was noticed. Then, the infusion was carried without the flow mesh as shown in Figure 7b to reduce the longitudinal resin flow rate. However, this attempt was not successful. The infusion was too slow and resin reached its gel time before the infusion was complete. Further, the panel was infused with reduced flow mesh length and resulted in much improved impregnation with only a small dry spot as shown in Figure 7c. The flow mesh length (per cent of the total laminate length) was varied from 50% to 80% to achieve the right balance between the preform filling and the impregnation time. Preform was better filled with reduced flow mesh lengths as compared to one with no and full flow mesh length. The laminate shown in Figure 7c was thin C-Ply™, (+45/−45)_9S_, of thickness 3.2 mm and was infused with reduced flow mesh length 80% at a single stage vacuum level of 400 mbar. Table 4 gives the details from the observations made on the infusion of abovementioned laminate with various flow mesh lengths. Only the preform infused with 80% flow mesh length showed satisfactory results with a very small dry spot. This led to the further need of optimising the vacuum level along with the reduced flow mesh length.

#### 3.2.3. Vacuum Levels

**Figure 8:**
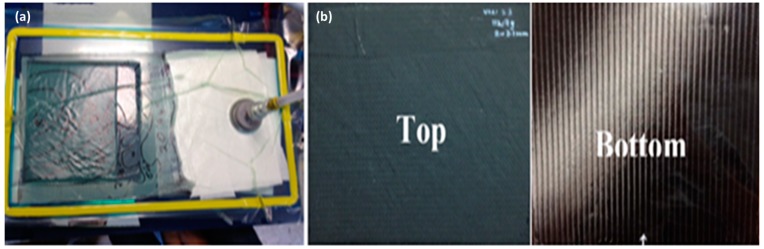
Infusion with optimised process parameters (**a**) set-up with reduced flow mesh length and three-stage vacuum level using glass mould; and (**b**) fully impregnated top and bottom sides of the panel.

Even though the thickness infusion was found to be significantly improved by reducing the flow mesh length, it was necessary to optimise the vacuum level and corresponding flow speed which has a detrimental effect on manufacturing of NCF laminates with low void content. Infusion of NCFs with both the matrices was carried out with one-, two-, and three-stage vacuum levels. Table 5 gives the details of the vacuum levels used in single and multi-stage phases of infusion process. Single stage infusion was carried out at similar infusion and consolidation vacuum levels. Two-stage infusion was used where first the resin was infused at low vacuum levels for balancing the impregnation in the longitudinal and transverse direction. Once the infusion was completed, the consolidation was carried out at higher vacuum level. In the case of three-stage vacuum level, the preform was infused with reduced flow mesh and with three different vacuum levels (lower vacuum during infusion on the flow mesh, low vacuum level beyond the flow mesh till end of infusion and high vacuum level for consolidation). A lower vacuum level was used for infusion at the start to prevent the resin from boiling and to ensure a slow infusion for good fibre impregnation. Once the infusion was complete, the vacuum level was increased to ensure better consolidation and increased V_f_. Figure 8a shows infusion of thin C-Ply™, (+45/−45)_9S_, panel of thickness 3.2 mm with a reduced flow mesh length and three-stage infusion while Figure 8b shows the fully injected top and bottom part of the same NCF panel.

### 3.3. Laminate Quality Optimisatin

VARI process optimisation was carried out to ensure complete impregnation of the thick and thin NCFs, as discussed in Section 3.2. It is necessary to investigate the quality of the manufactured panels as the void and porosities deteriorate the mechanical performance of the composite. Therefore, study was carried out to optimise the process parameters based on the final void content of the laminates.

#### 3.3.1. Effect of Single and Multi-Stage Vacuum Levels

##### Woven Fabrics

Usually, the infusion process for woven fabrics is easier, but still there was a need to optimise the infusion process parameter with Elium^®^ 280 resin. Infusion was carried out with the glass weave (200 mm × 200 mm) and Elium^®^ 280 resin at 31.3 mbar vacuum. The infusion was completed in 2.5 min, but with porosities of 3.69%. Although the bagging has passed the leak test, a substantial void content still arose due to the boiling of the resin when it reached the exotherm due to near maximum vacuum used during the process cycle. This is in general the tendency for the reactively processed resins and was found similar for the Elium^®^ 280 resin [31]. Thus, the vacuum was lowered to 330 mbar and the infusion completed in 5 min. The void content of this laminate was very low (0.88%). Hence, a single stage, 330 mbar was deduced as the optimum vacuum level to successfully inject woven fabrics with TP and TS matrices. Three laminates were manufactured with the optimised parameters. The coefficient of variation (CV) in the void content of these laminates was 2.3%.

##### NCFs

**Figure 9:**
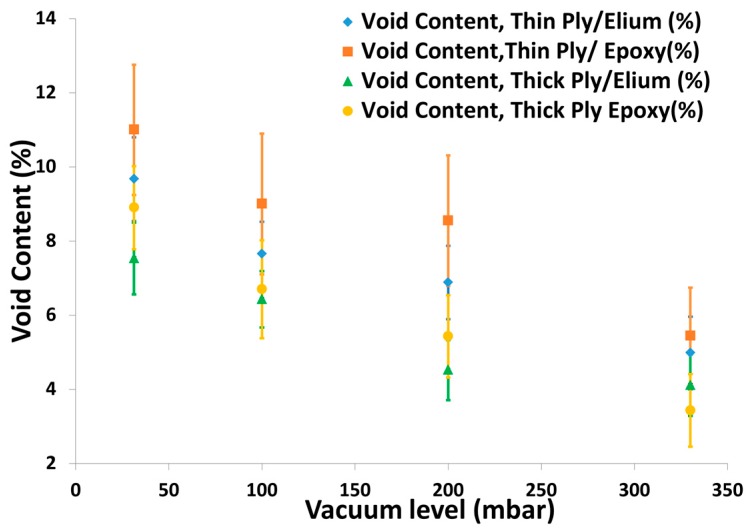
Void Content vs. vacuum level for thick and thin C-Ply™ with TP and TS matrices.

Thick and thin NCFs were injected with both TP and TS matrices. The fabric preform was injected with strong vacuum (31.3 mbar) and resulted in much higher void content with both the matrices, as seen from the Figure 9. Vacuum was further lowered to 100 mbar and 200 mbar with an aim to slow down the longitudinal infusion and give sufficient time for the resin to flow in the thickness direction. However, even with higher permeability thick NCF, a dry spot was still noticed on the bottom face of the laminate and resulted in larger void content. It was noticed that the resin flew quickly in the longitudinal direction while the bottom was not fully infused due to the flow mesh on the top and the lower transverse permeability of the NCFs [24,38,39]. Thick and thin NCFs were then injected with both the matrices using the optimised vacuum of 330 mbar established from woven fabrics infusion. Still a dry spot was found at the bottom face of the laminate resulting in a higher void content (see Figure 9).

**Figure 10:**
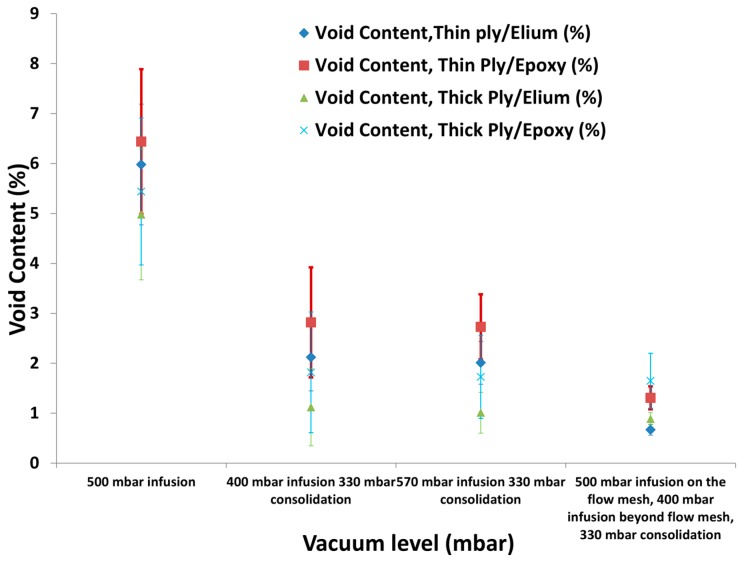
Void contents at one-stage, two-stage and three-stage vacuum level for thick and thin C-Ply™ with TP and TS matrices.

Then, the conceptualised two-stage vacuum was used to inject thin C-Plies with both the matrices, where first the preform was injected at lower vacuum level to slow down the longitudinal flow front and provide sufficient time for through the thickness infusion. However, the consolidation was carried out at a high vacuum level to achieve a higher fibre volume fraction. As can be seen from Figure 10, two-stage vacuum level where 400 mbar and 570 mbar vacuum was used for infusing thick plies resulted in much improved quality of laminate in terms of void content. Three sets of laminates were injected for each instance for checking the reliability of the results.

However, the results for the thin NCFs as seen from Figure 10 were still not satisfactory with two-stage vacuum levels with noticeable void content, and a larger coefficient of variation was observed. Thus, an optimised three-stage vacuum level with variable flow mesh length was established to infuse thin ply laminates with much improved quality as discussed in the next sub section.

#### 3.3.2. Effect of Flow Mesh Length Combined with Multi Stage Vacuum

**Figure 11:**
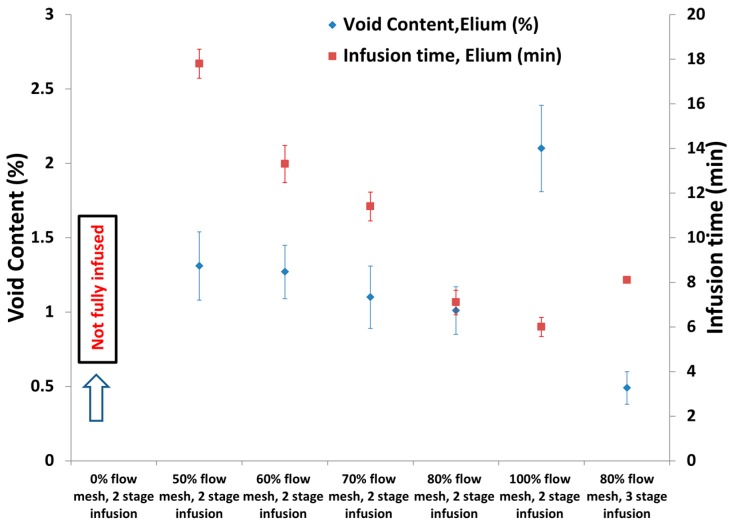
Void Content and infusion time at two-stage and three-stage vacuum level with varied flow mesh length for thin C-Ply™ with TP matrix.

It was noticed that only the vacuum does not play a crucial role in the successful infusion of the NCF laminate rather there is a need to play around with the material constraints especially flow mesh which increases the flow front speed considerably. Hence, the flow mesh length was varied from 50% to 80% of the total laminate length to slow down the infusion in longitudinal direction. With much lower flow mesh length (<50% of the total laminate), the infusion was possible, but was very long and was not a preferred solution. In addition, there is a constraint of resin gel time, which cannot be overlooked. Figure 11 shows the results for Elium^®^ 280 resin infusion with thin NCFs with different flow mesh length at two-stage infusion (570 mbar for infusion, 330 mbar for infusion). Considerably lower void content was noticed with 80% flow mesh length with two-stage infusion process compared to the one with other lower flow mesh dimensions. Ordinates in Figure 11 show the void content and the infusion time of the thin NCF with TP resin corresponding to various flow mesh lengths and the infusion levels. Infusion time increased with the reduced flow mesh length. Noticeably, the two-stage vacuum level with various flow mesh lengths resulted in improved quality of the panel compared to the single stage infusion, as seen from Figure 10. The infusion with two-stage vacuum level was found sufficient for thick plies, but, with thin plies, void content >2% along with larger scattering from the average values in the results was noticed. As the thin NCF laminates were still found to be with little entrapped air with two-stage infusion, the infusion was further slowed down on the flow mesh and three-stage vacuum levels with 500 mbar for infusion until the flow mesh followed by an increased vacuum level of 400 mbar beyond the flow mesh was adopted. Once the infusion was found completed from the mirror tracing the flow evolution below the glass plate, the laminate was consolidated at 330 mbar. This three-stage vacuum with 80% flow mesh length resulted in the laminate with minimal void content of 0.5% with low coefficient of variation. Epolam 5015/5015 epoxy and Elium^®^ 280 were successfully infused using these optimised infusion process parameters and at least 10 laminates were duplicated for both TP and TS panels for checking the reliability of the process optimisation.

### 3.4. Discussion on Optimised Parameters

#### 3.4.1. Deduced Scheme for Infusing NCFs

**Figure 12:**
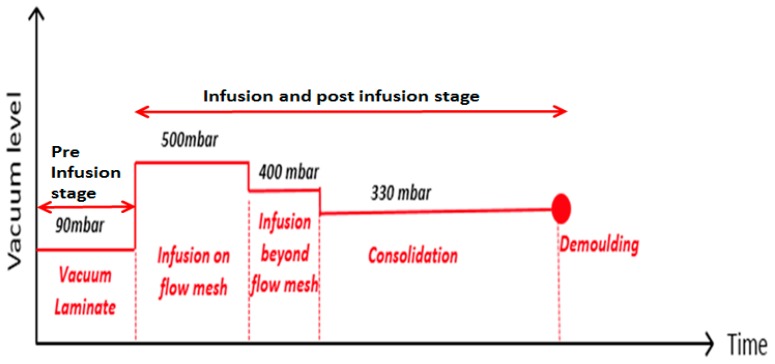
Optimised Three Stage vacuum levels deduced from VARI process and NCF composite quality optimisation.

Based on the optimised parameters of VARI processing and the quality of the manufactured NCF laminates with TP and TS matrices, deduced scheme is summarised below:

- Vacuum level: 500 mbar should be used for infusion on the flow mesh; increased to 400 mbar from end of flow mesh until the infusion was completed; and 330 mbar should be used for final laminate consolidation (see Figure 12).
- Infusion speed: Flow speed should be 15 cm/min on the flow mesh and then should be slowed down to 0.5 cm/min until the end of the laminate.
- Flow mesh: Length was reduced to 80% of the panel length to slow down the resin front in order to infuse properly in through the thickness direction, especially in the case of thin NCF.

#### 3.4.2. Consistency and Quality of NCF Laminates

**Figure 13:**
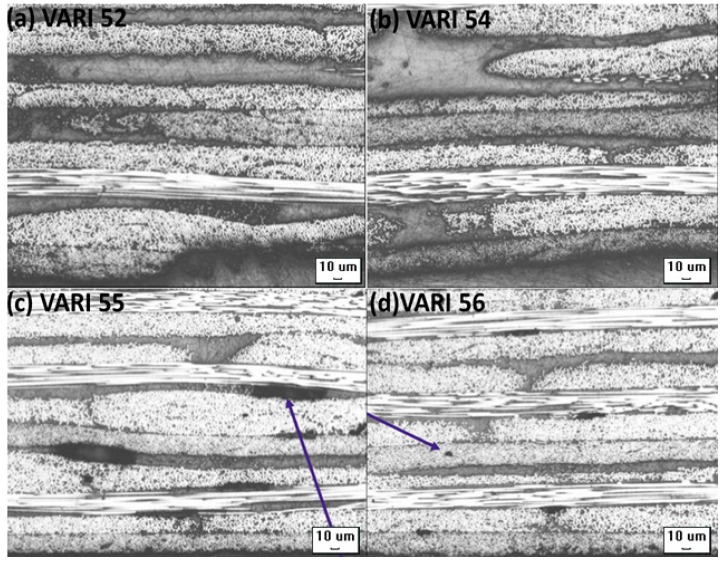
Micrographs showing the voids/porosities in various laminate configurations: (**a**) VARI 52 with no porosities; (**b**) VARI 54 with no porosities; (**c**) VARI 55 with macroporosities; and (**d**) VARI 56 with micro and macro porosities (all micrographs ×5 magnification).

Panels with thicknesses up to 4.2 mm, (+45/−45) ns, (0/90) ns, and (0/45/90/−45) ns orientations, up to 300 mm × 480 mm dimensions and with the room temperature cured resins were successfully manufactured using the optimised process parameters. The successive preform layers with large angular difference significantly increase the permeability of the fabrics [40] Preforms with the above-mentioned fabric orientations were injected to consider the effect of the ply orientation in through the thickness permeability of the fabrics. It was noticed that quasi-isotropic laminate was easier and faster to inject compared to the laminate of (0/90) ns layup having similar thickness and the same number of layers. The consistency achieved in the laminate manufacturing of thick and thin NCFs with TP and TS matrices is presented in Table 6. VARI 52 and 54 were carried out with the thin and thick NCFs respectively with Elium^®^ 280 resin. Both the laminates were free from porosities with a void content of only (0.1%) was observed as can be seen from Figure 13a,b. The infusion time was 21.5 min for thin NCF while the thick NCF panel with Elium^®^ 280 resin was injected in 6.2 min only owing to the higher permeability of the thick plies. VARI 55 and VARI 56 were injected with epoxy resin with the same optimised parameters and the laminates with considerably higher volume fraction were achieved. Although there were micro and macro porosities present in the laminate samples (see Figure 13c,d), they were within the acceptable range considering there were no substantial pressure as well as heat cycle involved in the curing process. VARI 73 was infused with preform of 300 mm × 480 mm dimension and was fully injected using the optimised process parameters.

### 3.5. Comparison of TP and TS Matrices

The comparison between Elium^®^ 280 and epoxy in context of their processing can be made based on the deduced results. As both the matrices have similar viscosities of 200 cP, there is no marked difference in the infusion time with thick and thin NCFs. Considering the room temperature cure manufacturing, Elium^®^ 280 has about 6 times faster demoulding time compared to epoxy resin in the present investigation. In addition, Elium^®^ 280 resin does not require significant degassing as 20–30 s mixing of powder based BPO is sufficient and saves additional 20 min of necessary degassing time required for epoxy resin. Being a reactive thermoplastic monomer, the major need of time is a precaution that should be taken to keep the fabrics and mould free from dust. In addition, the moisture should be controlled to avoid the significant chances of deactivating the initiator. It should be noted that Elium^®^ 280 resin is available in various formulations (viscosity and pot life) and the infusion and demoulding time can be further reduced with introduction of heat. Elium^®^ 280 can be cured in as fast as 6 min with mould temperature at 80 °C. Considering the 24 h curing time of epoxy resin with continuous vacuum, the laminates resulted in slightly higher fibre volume fraction compared to Elium^®^ 280 composites. In addition, the void content in the NCFs composites with Elium^®^ 280 was comparatively lower than epoxy based composites.

## 4. Conclusions

The present research gives the details of the process parameters for manufacturing thick and thin carbon NCFs with epoxy and liquid Methyl methacrylate (MMA) thermoplastic resin using VARI process. Infusion parameters were essentially required for optimisation, especially for injecting new thermoplastic resin with low permeability thin NCFs. After several iterations, it was found that using a three-stage vacuum for infusion and consolidation contributed to manufacturing panels with consistent fibre volume fractions with minimal void content. It was also found out that optimal flow mesh length of 80% of the panel length slowed down the resin front in order to infuse properly in through the thickness direction, especially in the case of thin plies. The optimised flow speed was found to be 15 cm/min on the flow mesh and then slowed down to 0.5 cm/min until the end of the laminate. Abovementioned optimised parameters lead to successfully inject thick and thin NCFs composites with greater consistency with void contents normally lower than 1% and higher fibre volume fractions of up to 62%. These findings are of significant importance, as they present a way to manufacture textiles composites that are largely used in major industries such as aerospace and automotive for mass production of structural composite parts.

## Figures and Tables

**Table 1:** Fabric Reinforcement used in current research.

	Glass Fibre	C PLY™	C PLY™	C PLY™	C PLY™	C PLY™	C PLY™
Orientations	Plain weave	0/90	+45/−45	0/+45	+0/−45	0/+45	+0/−45
Individual ply areal weight (g/m^2^)	200	200	74	100	100	75	75
Total weight (g/m^2^)	200	400	151	200	200	150	150
Sizing	-	TP	TS	TP/TS	TP/TS	TP/TS	TP/TS

**Table 2:** Properties of chosen resin systems in current research.

Resin	Hardener/Initiator	Mixing Ratio	Density (g/cm^3^)	Tensile Modulus (GPa)	Tensile Strength (MPa)	Tensile Elongation (%)	T_g_ (Glass Transition Temperature)/°C	Fracture Toughness, G_1c_ (kJ/m^2^)
Elium^®^ 280	Benzoyl Peroxide	3% with resin	1.2	3.3	76	6	120	0.5
Epolam 5015	5015	70:30	1.1	3.1	80	7	85	0.12

**Table 3:** Permeability values (V_f_ = 51%–52%) for thick and thin NCFs.

Fabric Type	K_x_ (m²)	K_y_ (m²)	K_z_ (m²)	Reference
Thin C-Ply^TM^ (200 g/m^2^)	1.27 × 10^−11^	1.1 × 10^−11^	2.1 × 10^−13^	Chomarat
Thick Seartex (322 g/m^2^)	2.1 × 10^−9^	8.7 × 10^−10^	-	[34]

**Table 4:** Effect of flow mesh length on preform infusion at single stage vacuum.

	Flow Mesh Length (Per Cent of Total Laminate Length)						
		0%	50%	60%	70%	80%	100%
Observations on preform filling at 400 mbar infusion	Top surface of laminate	Cannot fully infuse (Figure 7a)	Small dry spot	Smaller dry spot	Small dry spot	No dry spot (Figure 7b)	No dry spot (Figure 7c)
	Bottom surfce of laminate	Cannot fully infuse (Figure 7a)	Massive dry spot	Massive dry spot	Small dry spot	Very small dry spot (Figure 7b)	Massive entrapped air (Figure 7c)
Quality		Not acceptable	Not acceptable	Not acceptable	Not acceptable	Acceptable	Not acceptable

**Table 5:** Vacuum levels used in single and multi-stage phases of infusion process.

Vacuum Functions	Vacuum Levels *		
	Single Stage	Two-Stage	Three-Stage
Evacuate trapped air (Pre infusion stage)	Highest	Highest	Highest
Infusion	Highest to High	Low to Lower	On flow mesh-Lower Beyond flow mesh-Low
Consolidation	Same as infusion	High	High

* Reference vacuum levels: Highest 31.3–90 mbar, Higher 90–150 mbar, High 150–350 mbar, Low 350–450 mbar Lower 450–600 mbar.

**Table 6:** Summary of various panels injected with optimised process parameters

Panel	VARI 52	VARI 54	VARI 55	VARI 56	VARI 73	VARI 78
FAW (g/m^2^)	200	400	200	150	400	200
Fibre type	C ply™	C ply™	C ply™	C ply™	C ply™	C ply™
Resin	ELIUM^®^ 280 + BPO	ELIUM^®^ 280 + BPO	EPOLAM 5015/5015	EPOLAM 5015/5015	ELIUM^®^ 280 + BPO	ELIUM^®^ 280 + BPO
Dry fibres weight (g)	105 ± 0.2	102.6 ± 0.2	105.1 ± 0.2	114.3 ± 0.2	159.4 ± 0.2	161.1 ± 0.2
Number of plies	8	4	8	12	4	8
Layup	(0/45/90/−45)_2s_	(0/45/90/−45)_s_	(0/45/90/−45)_2s_	(0/45/90/−45)_3s_	(0/90)_2S_	(0/45/90/−45)_2s_
Size (mm×mm)	250 × 50	250 × 250	250 × 250	250 × 250	300 × 480	300 × 480
Infusion time (m)	20.5	6.2	21	21	26.1	33
Vacuum level (mbar)	500 and 400 infusion 330 consolidation	500 and 400 infusion, 330 consolidation	500 and 400 infusion, 330 consolidation	500 and 400 infusion, 330 consolidation	500 and 400 infusion, 330 consolidation	500 and 400 infusion, 330 consolidation
Mass after infusion (g)	157.1 ± 0.4	153.7 ± 0.4	145.5 ± 0.4	162.8 ± 0.4	253.5 ± 0.4	255.3 ± 0.4
Thickness (mm)	2.12	2.08	2.17	2.11	2.07	2.02
Quality/Void Content (%)	0.1	0.1	1.31	1.08	0.83	0.77
Fibre mass Fraction (%)	66 ± 0.1	66 ± 0.1	72 ± 0.1	70 ± 0.1	62.80 ± 0.1	63.1 ± 0.1
Fibre volume Fraction (%)	56.90 ± 0.2	56.80 ± 0.2	61.30 ± 0.2	59 ± 0.2	52 ± 0.2	52.8 ± 0.2
